# Leveraging genome-wide datasets to quantify the functional role of the anti-Shine–Dalgarno sequence in regulating translation efficiency

**DOI:** 10.1098/rsob.160239

**Published:** 2017-01-18

**Authors:** Adam J. Hockenberry, Adam R. Pah, Michael C. Jewett, Luís A. N. Amaral

**Affiliations:** 1 Interdisciplinary Program in Biological Sciences, Northwestern University, Evanston, IL 60208, USA; 2 Department of Chemical and Biological Engineering, Northwestern University, Evanston, IL 60208, USA; 3 Northwestern Institute on Complex Systems, Northwestern University, Evanston, IL 60208, USA; 4 Kellogg School of Management, Northwestern University, Evanston, IL 60208, USA; 5 Chemistry of Life Processes Institute, Northwestern University, Evanston, IL 60208, USA; 6 Department of Physics and Astronomy, Northwestern University, Evanston, IL 60208, USA

**Keywords:** translation initiation, translation efficiency, gene expression

## Abstract

Studies dating back to the 1970s established that sequence complementarity between the anti-Shine–Dalgarno (aSD) sequence on prokaryotic ribosomes and the 5′ untranslated region of mRNAs helps to facilitate translation initiation. The optimal location of aSD sequence binding relative to the start codon, the full extents of the aSD sequence and the functional form of the relationship between aSD sequence complementarity and translation efficiency have not been fully resolved. Here, we investigate these relationships by leveraging the sequence diversity of endogenous genes and recently available genome-wide estimates of translation efficiency. We show that—after accounting for predicted mRNA structure—aSD sequence complementarity increases the translation of endogenous mRNAs by roughly 50%. Further, we observe that this relationship is nonlinear, with translation efficiency maximized for mRNAs with intermediate levels of aSD sequence complementarity. The mechanistic insights that we observe are highly robust: we find nearly identical results in multiple datasets spanning three distantly related bacteria. Further, we verify our main conclusions by re-analysing a controlled experimental dataset.

## 1. Introduction

The abundance of different protein species within a single cell can vary by several orders of magnitude, and multiple points of control are critical for tuning the expression of individual proteins over such a wide range [1–4]. Transcription of the gene of interest is a necessary first step in the pathway of gene expression but, by itself, transcription is insufficient to ensure protein expression; studies in a variety of organisms have shown that mRNA abundances only modestly predict protein abundances [4–9]. The magnitude of these correlations remains open to debate, and part of the lack of a strong relationship between mRNA and protein abundances is probably a result of differential protein degradation rates and noisy measurements of both quantities [10]. It is, however, clear that the rate at which different mRNA species are translated into their protein product is variable and may be a significant source of variation in protein abundance and a point of regulation [3,11].

In studies dating back to the 1970s, researchers noted that a thermodynamic interaction between the 16S ribosomal RNA and the 5′ untranslated region (UTR) of mRNAs is important for overall translation efficiency—defined here as the number of protein molecules made per mRNA per unit time—by enhancing translation initiation in prokaryotes [12]. The strength, optimal distance to the start codon and structural accessibility of this anti-Shine–Dalgarno::Shine–Dalgarno (aSD::SD) sequence interaction all play a crucial role in modulating the rates of translation initiation and thus protein abundances [13–17]. More recently, multiple studies have reinforced this paradigm and continue to elucidate the finer details about the importance of translation initiation signals, highlighting the fact that surrounding nucleotides may constrain SD sequence evolution owing to mRNA structural constraints [18–23].

Much of our understanding about the process of translation initiation has come from experimental researchers expressing multiple genetic constructs with slightly varying 5′ UTRs placed upstream of a heterologous gene whose output is easy to quantify. However, most studies have looked at a relatively small number of such easily quantifiable genes that have been expressed in a small subset of experimentally tractable species, often at high levels. Experimental studies present a well-controlled system to interrogate these mechanisms, but the degree to which these findings can be extrapolated more broadly to different genes, species and expression levels remains largely unknown. Nevertheless, researchers' ability to predict translation rates of heterologous genes have continually improved as more and more detailed experimental data are generated and incorporated into biophysical models [3,19].

**Figure 1.**
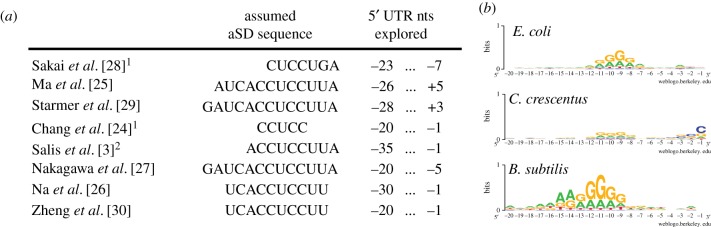
SD sequence usage is variably defined in the literature and differs between genomes. (*a*) Several studies report a range of relevant parameters used to identify the aSD::SD sequence interaction. (^1^denotes studies that implicitly derive aSD sequences by extrapolating from over-represented UTR motifs; ^2^denotes studies that explicitly penalize for non-optimal distances to the start codon). (*b*) Sequence logos demonstrate that 5′ UTRs are highly non-random within a given species, largely a result of significant purine enrichment. However, the magnitude of this enrichment and the spacing relative to the start codon vary between species despite widespread conservation in the 3′ end of the 16S rDNA.

In parallel, a number of different studies have analysed various facets of translation initiation sequence variation across bacteria using bioinformatic or computational means, but definitions about which genes to consider as ‘SD genes’ vary broadly [3,24–30]. The main differences frequently concern where to look upstream of the start codon for a putative SD sequence and what bases of the 16S rRNA sequence to consider as the aSD sequence when assessing sequence complementarity to the 5′ UTR of mRNAs (figure 1*a*). Despite their differences, bioinformatic investigations have consistently shown that SD sequences occur much more frequently than random expectation in the 5′ UTRs of most species, further suggesting a large role for aSD sequence complementarity in regulating translation initiation (figure 1*b*).

Finally, as genome-scale and high-throughput sequencing technologies have come of age, a third route of investigation has become possible. By measuring the translational status of thousands of different genes within a single experiment, ribosome profiling (Ribo-seq) and RNA sequencing (RNA-seq) technologies can be combined to allow researchers to determine translation efficiencies across the genome [31]. Application of this technique to multiple organisms has already enhanced our understanding of translational regulation, stoichiometric protein production, determinants of elongation speed and genome annotation [11,31–33]. However, in the context of bacterial translation initiation, several studies have suggested that the aSD binding strength shows no discernible relationship with the measured translation efficiency of endogenous genes at the genome scale [11,33,34]. The negative results of these studies may be due to a variety of non-mutually exclusive factors, including (i) noisy or inaccurate estimates of translation efficiency from these data, (ii) suboptimal parameters associated with assessing the aSD sequence relationship, (iii) difficulty accounting for the effect of mRNA structures surrounding the start codon through computational means, (iv) the fact that many endogenous mRNAs are translationally regulated or present in operons, and, finally, (v) the lack of a relationship in these data may be real—requiring researchers to re-think our understanding of the mechanisms governing translation initiation in bacteria.

Here, we investigate whether the sequence diversity of endogenous genes can be leveraged along with ribosome profiling-based estimates of translation efficiencies to precisely define the relevant parameters associated with aSD::SD sequence interaction. Rather than attempt to develop a comprehensive model to explain as much of the variation in translation efficiencies as possible, we instead propose a simpler question: can empirically measured translation efficiencies help us to better understand the particular phenomenon of aSD sequence complementarity and its role in regulating translation efficiencies? Our data-driven analysis yields definitions for the optimal distance between predicted aSD sequence binding and the start codon, and the extents of the aSD sequence itself. We further highlight a highly conserved nonlinear relationship between aSD sequence complementarity and translation efficiency of endogenous genes whereby intermediate complementarity maximizes translation efficiency downstream genes. We confirm these findings in multiple independent genome-scale and experimental datasets, and in doing so highlight the robustness of our conclusions while validating that the size of this effect is greatly enhanced as experimental steps are taken to reduce error in translation efficiency measurements.

## 2. Results

### 2.1. Deriving translation efficiency measurements from Ribo- and RNA-seq

For a given mRNA, ribosome density maps derived from ribosome profiling can be used to illustrate regions of relatively fast and slow translation. When used in conjunction with RNA-seq to estimate mRNA abundances, this ground-breaking technology allows researchers to roughly quantify relative translation efficiency (RTE) on a per gene basis for thousands of genes in a single experiment. However, it is important to note that estimates of RNA abundances and ribosome occupancies are both error-prone owing to biological noise as well as the numerous steps in the experimental process that may introduce systemic bias [35–39]. Thus, RTE is a particularly noisy approximation, because error is compounded when dividing two error-prone values. We therefore established several quality controls for gene inclusion that are stricter than those previously used in the literature (see Materials and methods). Following on the previous work of others [11,33], we then calculated RTE per gene as2.1
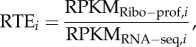
where 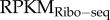 and 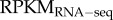 are reads per kilobase per million mapped reads (RPKM) for a gene, *i*, obtained through ribosome profiling and RNA-seq, respectively. Using the original Ribo- and RNA-seq mappings provided by three separate studies in rich media for *Escherichia coli*, *Caulobacter crescentus* and *Bacillus subtilis* we derived measurements of translation efficiency for 2910, 1833 and 2385 genes, respectively (electronic supplementary material, figure S1) [11,33,40]. While this metric relies on some crucial assumptions, such as equivalent elongation rates between genes, prior work has shown that these assumptions are generally valid [11]; a noise-free *RTE* metric calculated in this manner should be highly correlated with ‘true’ translation efficiencies as we have defined it. We note that we investigated several variations in the above metric (such as excluding the beginning and the end of genes, Winsorizing to limit extreme values, removing the lowest mRNA expression decile, etc.), but none of these variations led to distinguishably different results so for the purposes of this manuscript we opt for the simplicity of equation (2.1) moving forward.

As others have noted, mRNA structure surrounding the start codon is known to influence translation initiation, perhaps playing a dominant role in determining translation efficiency [2,11,16,21,41]. We confirmed this finding by showing that log-transformed translation efficiencies in all three organisms showed highly significant correlations with the predicted degree of mRNA secondary structure (Δ*G*_folding_) in the initiation region (defined here as −30 to +30 nucleotides relative to the first base of the start codon, which was labelled +1; *R*^2^ = 0.13, 0.10 and 0.08 for *Escherichia coli*, *C. crescentus* and *B. subtilis*, *p* < 10^−42^ for all cases). Given the strength of this correlation (electronic supplementary material, figure S2), we analyse the residuals from this predictive model (in units of log-scaled translation efficiency) in order to determine what role, if any, aSD sequence complementarity has in modulating translation efficiency2.2
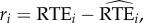
where RTE_*i*_ is the relative translation efficiency of gene *i*, and 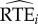 is the estimate of RTE for gene *i* derived from the regression on Δ*G*_folding_ for each dataset. Put more simply, the residual RTE value for a gene is the difference in observed RTE minus the predicted RTE where our prediction is based off of the mRNA structure. We include this step to alleviate the source of biological variation associated with *cis*-structure, but note that these computational predictions also introduce error due to the—at best—modest correlation between computationally predicted structures and their counterparts as they exist *in vivo* [42]. Later, we show that all of our primary results remain significant, albeit with decreased magnitude when we skip this step and instead investigate RTE values directly.

### 2.2. Defining the optimal distance to the start codon and species specific aSD sequences

**Figure 2.**
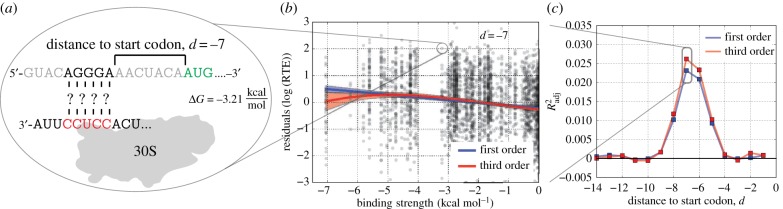
Determining the optimal distance to the start codon. (*a*) Illustration of the method used in this study for determining the predicted Gibbs free-energy (Δ*G*_binding_) of the hybridization of the putative aSD sequence (highlighted in red) to the five-nucleotide sequence at a distance of seven nucleotides upstream from the start codon. (*b*) The strength of aSD binding for each gene at a distance of −7 is correlated against the model residuals in units of 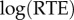. Shown are first- and third-order polynomials (*R*^2^_adj_ = 0.023 and 0.026, respectively, *p* < 10^−16^ for both). (*c*) We performed the same correlation analysis as in (*b*) for each putative distance to the start codon in the *E. coli* dataset for the given aSD sequence. Shown are the *R*^2^_adj_ values for the relevant models with a maximum peak for *d* = −7.

Using the residual RTE values described in equation (2.2), we took a systematic approach in order to determine where to look, in an unbiased manner relative to the start codon, for the statistical signal of aSD sequence complementarity under the assumption that the true value of this parameter should show the strongest correlation between aSD sequence complementarity and residual RTE values. For each gene, we calculated the predicted hybridization energy of the core aSD sequence (5′-CCUCC-3′) to each sequential 5-mer upstream of the start codon (figure 2*a*). Hereafter, we refer directly to the location (relative to the start codon) as the number of bases between the fragment analysed and the start codon (this metric of distance corresponds to the aligned spacing presented by Chen *et al.* [14]). We asked how well the aSD sequence complementarity at a particular location for all genes performed at predicting residual *RTE* values via both linear and third-order polynomial regression.

In figure 2*b* we show example data for a distance to the start codon of −7 nucleotides (assessing complementarity of nucleotides −12 through −8 for each gene). We show both the first- and third-order fits for the residual *RTE* data from *E. coli*, and find that both correlations are small yet highly significant (*F*-test, *p* < 10^−16^). Further, in figure 2*c* we show the adjusted-*R*^2^ (*R*^2^_adj_) resulting from repeating the correlations shown in figure 2*b* for each indicated distance relative to the start codon. We use the *R*^2^_adj_ metric hereafter, because unlike *R*^2^ this adjusted metric penalizes for increasing parameter numbers associated with more complex third-order polynomial models and thus helps guard against over-fitting to the data. Despite the relatively small *R*^2^_adj_ values, the sharpness of this peak shows that there is a clear and highly significant relationship between aSD sequence complementarity in the 5′ UTR of mRNAs and translation efficiency. The third-order polynomial model was slightly more predictive at this stage, so we present our data in the form of third-order polynomial regressions hereafter except where otherwise noted.

**Figure 3.**
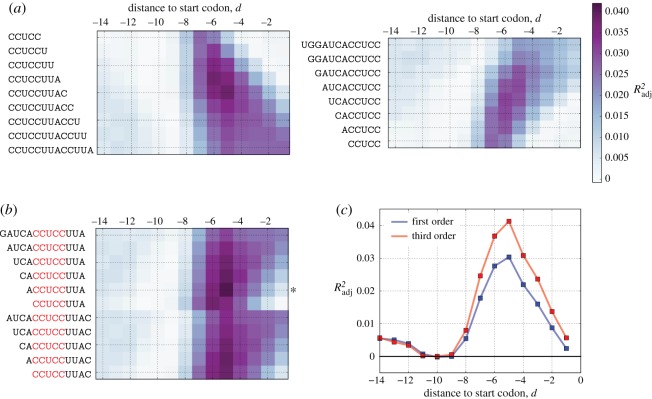
Parameter fitting landscape to determine optimal aSD and distance values. (*a*) *R*^2^_adj_ from the third-order model at different distances to the start codon and various 3′ and 5′ extensions to the core aSD for *E. coli*. (*b*) Combination of best-fitting putative aSDs from (*a*) to determine the optimal aSD sequence and distance parameters based on their fit to the residual RTE data (asterisk denotes the selected best-fitting aSD sequence). (*c*) Comparison of *R*^2^_adj_ between the first- and third-order polynomial models from the best-performing aSD sequence from (*b*).

Our choice of 5′-CCUCC-3′ as the aSD sequence in figure 2 was simply to illustrate our methodology by using the most conserved region of the 16S rRNA tail. In practice, it is not clear precisely which 16S bases belong to the aSD sequence although the 3′ tail of *E. coli* has been experimentally determined to end with 5′-…CCUCCUUA-3′. In order to see if the data would allow us to recover the expected aSD sequence, we repeated the above analysis for different putative aSD sequences extending in the 5′ and 3′ directions at different binding locations and observed increasing *R*^2^_adj_ values and a slight re-positioning of the optimal distance to the start codon (figure 3*a*). It should be noted, however, that this change in the optimal distance is partially an artefact of our numbering scheme. As we include more 5′ bases in the definition of the aSD sequence, even if the location of optimal binding for a given mRNA does not change, the ‘distance’ will change based on the fact that it is calculated relative to the 5′ end of the putative aSD sequence (electronic supplementary material, figure S3). In this analysis, we extend past the known rRNA sequence tail as a control that will allow us to test the accuracy of our method by determining whether it is able to uncover the known 3′ terminus.

We finally explored a range of variants that include extensions on both ends to determine the optimally predictive aSD sequence and distance parameters for the given dataset (figure 3*b*). Several of these putative aSD sequences produced similar results, so we selected the shortest sequence among these candidates (5′-ACCUCCUUA-3′), but we stress that our methodology can probably not discriminate these boundaries precisely given the small differences in *R*^2^_adj_ values between putative aSDs with single base additions/deletions. While the overall correlation coefficient in this best-fitting model is still modest (*R*^2^_adj_ = 0.041), the significance of this finding is extremely high (*p*<10^−26^), indicating that despite the potentially large error in *RTE* estimates, we are nevertheless able to observe a highly significant underlying relationship. These data further show that although complementarity to the core aSD sequence shows a roughly linear relationship with RTE (the third-order model in figure 2*c* performs only slightly better), the inclusion of flanking sequences results in both increasing predictive power as well as increasing nonlinearity in the underlying relationship. Finally, as a further indicator of the accuracy of this method, it resulted in a frequently cited aSD sequence of 5′-ACCUCCUUA-3′, thus uncovering the experimentally determined 3′ terminus.

### 2.3. The relationship between aSD binding and translation efficiency

In order to test the generality of our findings for *E. coli*, we next tested whether our methodology could produce comparable results for *B. subtilis* and *C. crescentus*. We found that the 5′ extensions are similar for the different organisms studied with *B. subtilis* showing preference for a slightly longer 5′ aSD extension, a finding that is consistent with prior observations that the canonical SD sequence in *B. subtilis* 5′ UTRs appears shifted further upstream of the start codon (figure 1*b*). We further found that species-specific 3′ extensions to the 16S rRNA result in enhanced correlations and thus are probably present in the processed 16S rRNA (to the best of our knowledge, the precise 3′ 16S rRNA terminus for these species is unknown) and participate in message discrimination for these two organisms (electronic supplementary material, figures S4 and S5). For *C. crescentus* the aSD sequence that we obtained from our data-driven model is 5′-CCUCCUUUC-3′ while for *B. subtilis* the corresponding sequence is the 5′ extended 5′-UCACCUCCUUUCUA-3′. However, as with *E. coli*, it is difficult to discern whether single base additions/deletions to the ends of these putative aSD sequences are functional.

**Figure 4.**
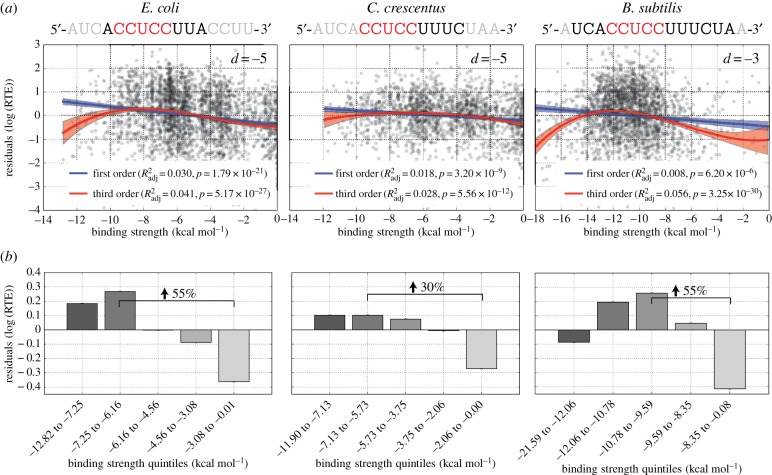
Summary of findings for three independent organisms using ribosome profiling-based data. (*a*) Scatter plot of residual RTE values after accounting for the effect of mRNA structure versus aSD sequence complementarity for the species-specific optimal aSD sequence (shown above in black) and optimal distance to the start codon (inset). (*b*) Data from (*a*) depicted as equally sized quintile bins to illustrate the magnitude of the effect. Bars denote the mean within each bin, whereas error bars show standard error of the mean. Percentage increase highlights the average increase in translation efficiency expected for a gene with aSD sequence complementarity at the optimal distance compared with a gene with weak aSD sequence complementarity.

Despite the vast evolutionary distance between these species, the functional form of the best-fitting models was highly similar for all three, showing the highest residual RTE values for intermediate binding strengths with similar predictive powers in the third-order model (*R*^2^_adj_ = 0.041, 0.028 and 0.056, for all cases *p* < 10^−11^; figure 4*a*). We further verified that nonlinear models provide a superior fit to the data—even though *R*^2^_adj_ explicitly punishes models with more parameters—via the Akaike information criterion (AIC), a stringent model selection metric used to judge the relative quality of model fits while explicitly penalizing for parameter number (electronic supplementary material, figure S6).

In order to more clearly show the magnitude of the observed effect—and for strictly illustrative purposes—we split the data for each organism into equally sized quintile bins (i.e. the 20% of genes with the highest aSD sequence complementarity, through to the 20% with the lowest). Notably, treating the data this way involves no model fitting, and in doing so we observe that (i) the average gene which binds the aSD sequence at the intermediate-to-strong binding strength level shows a 30–50% increase in translation efficiency compared with an average gene that binds the aSD very weakly (figure 4*b*) and (ii) the strongest binding quintile of genes exhibits either decreased or equivalent translation efficiency compared to the bin with intermediate-to-strong aSD binding strength. This suggests that mRNAs that contain sequences that bind too strongly to the aSD sequence may actually show reduced translation efficiency, a point that has support from several prior studies in the literature working with experimental systems [43,44]. We note, however, that the optimal sequence complementarity bin for *B. subtilis* is larger than the optimal bin for *E. coli* and *C. crescentus*. This variation may be a result of true underlying differences between the translation initiation mechanisms between these distantly related species, or a function of the fact that the *B. subtilis* aSD sequence is much longer, resulting in a broader range of sequence complementarity values than is observed for the other species.

To test the robustness of the above findings to some of our previous assumptions, we repeated the analysis from figures 3 and 4 by interrogating log-transformed RTE values directly. Although *cis*-mRNA structure is thought to be an important regulator of translation initiation, we are faced with the reality that our computational predictions of structural stability are rough approximations of *in vivo* structures, and therefore may introduce further error and biases into our measurements. Nevertheless, we observed very similar results for all three organisms in terms of the optimal aSD sequence and distance (electronic supplementary material, figure S7) as well as the functional form of the best-fitting model (electronic supplementary material, figure S8). The fact that the significance of our results is improved when removing the effect of mRNA structure provides further evidence that the *true* magnitude of the aSD sequence complementarity effect may be even further enhanced were we able to more accurately predict—and control for—the structural component of this relationship.

**Figure 5.**
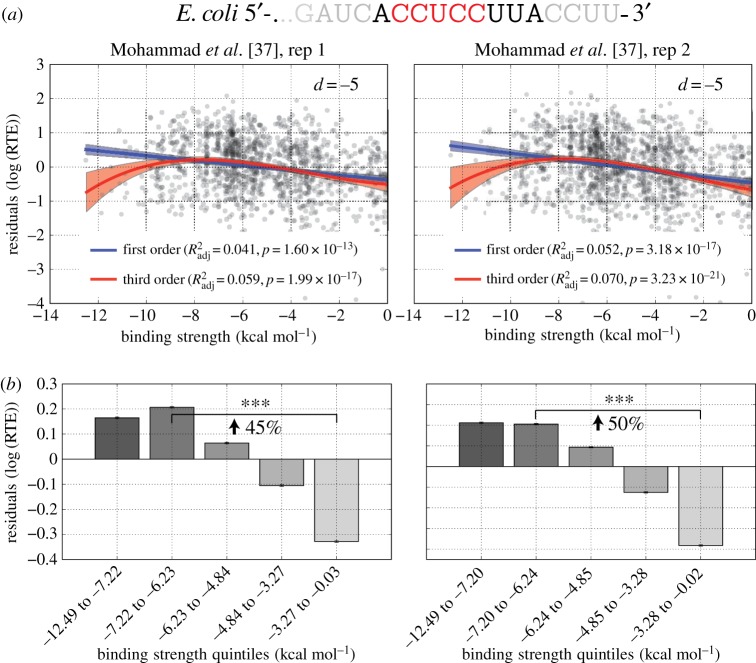
(*a*,*b*) Validation of findings in independent *E. coli* ribosome profiling datasets. Scatter plot and quintile analysis for independent *E. coli* datasets as in figure 4. For both replicates, the optimal fitting aSD sequence and distance to the start codon were the same as shown in figure 4 for *E. coli* with largely similar trends and stronger correlation, presumably owing to a reduction in measurement error.

Given recent concerns in the literature about the possibility of biases arising from the size selection step of prokaryotic ribosome profiling studies, we analysed two further *E. coli* datasets (*n* = 1278 and 1321) from an independent laboratory that were generated in such a way as to purportedly minimize potential sources of error [37]. After accounting for mRNA structure as before (*R*^2^ = 0.11, *p*<10^−33^ for both datasets), we observed nearly identical results to the previous *E. coli* dataset (figure 5; electronic supplementary material, figure S6). For both replicates, the 5′-ACCUCCUUA-3′ aSD sequence at a distance of −5 provided the best fit to the data, with corresponding *R*^2^_adj_ values of 0.06 and 0.07 for the best-fitting third-order polynomial and effect sizes of 45% and 50%. While illustrating the robustness of our results for a given organism across multiple independent datasets, this analysis also highlights the sensitivity of *R*^2^_adj_ to measurement noise. Although we observed generally low, albeit highly significant, *R*^2^_adj_ values in the previous analyses, we saw a 50% increase in predictive power using the same modelling approach when applied to these new data while the effect size remains relatively insensitive to this scatter. Indeed, in these data, the correlation between aSD sequence complementarity and residual RTE is nearly as large as the correlation between mRNA structure and RTE supporting previous observations of a strong role for the aSD sequence in enhancing translation initiation.

Finally, given the propensity of prokaryotic genes to occur in operons, we repeated our analysis for all five datasets (using the previously discovered organism specific aSD and distance parameters) by splitting genes up according to whether they are predicted to be first in a transcription unit or in the middle/end (see Material and methods). Our results were variable for the different organisms, with our model-fitting procedure resulting in substantially increased predictive power for genes in the middle/end of operons for the *E. coli* datasets, whereas the opposite phenomenon was evident in the *C. crescentus* and *B. subtilis* data (electronic supplementary material, figure S9). Nevertheless, all correlations were highly significant and the third-order polynomial model—having a maximum value for intermediate aSD sequence complementary—resulted in larger *R*^2^_adj_ values compared with linear models for all datasets, further illustrating the robustness of this finding.

### 2.4. Translation efficiency in other datasets

**Figure 6.**
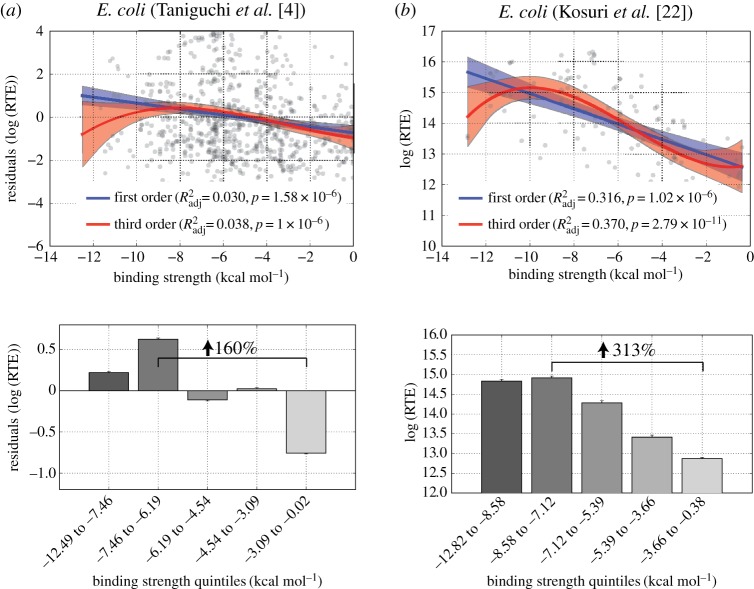
Validation of principal findings in non-ribosomal profiling based datasets. (*a*) Genome-wide data from Taniguchi *et al.* [4] show a significant relationship between aSD binding strength and residual RTE values. Quintile analysis shows a 160% increase in RTE between genes with weak and intermediate-to-strong aSD sequence complementarity. (*b*) Experimental data from Kosuri *et al.* [22] show the same trend as in figure 4 (*R*^2^_adj_ = 0.32 and 0.37 for first- and third-order models, *p*<10^−10^ for both cases). Quintile analysis shows a large effect size as well as a plateau or slight decrease for the quintile with the largest degree of aSD sequence complementarity.

To make sure that our observations are not a result of unknown systemic bias in the ribosome profiling-based method of calculating RTE, we turned to two separate datasets. First, we used an independent dataset from Taniguchi *et al.* [4], who estimated protein production per mRNA from the green fluorescent protein (GFP)-tagged single-cell protein distributions for 1018 *E. coli* genes (see Materials and methods for our quality control procedures) [4]. Using their data, we performed the same analysis as above and again observed nearly identical results to those seen in figure 4 for *E. coli*. In other words, the data exhibit a maximum at intermediate-to-strong aSD sequence complementarity (figure 6*a*; electronic supplementary material, figure S10). When we limit our analysis of this dataset to genes with the highest signal-to-error ratio (specifically, the top 50% as calculated by Taniguchi *et al.* [4]), the magnitude of the *R*^2^_adj_ gets larger with 5′-ACCUCCUUA-3′ sequence complementarity at a spacing of −5 predicting residual RTE with an *R*^2^_adj_ of 0.075 (*p* < 10^−6^) (electronic supplementary material, figure S10).

Finally, although our interest here is in the relationship between aSD sequence complementarity and the translation efficiency of endogenous genes, we further verified our main conclusions using a controlled experimental dataset [22]. Kosuri *et al.* [22] measured the strength of 111 ribosome binding sites (RBS) by creating synthetic constructs whereby RBS/promoter combinations drove expression of a downstream GFP reporter (see Material and methods). For each RBS, the protein produced per mRNA, averaged across the different promoter constructs, is an indicator that we will again refer to as RTE for simplicity. For these data, we did not remove the effect of mRNA structure, because each RBS data point represents an average across multiple independent mRNA species (derived from different upstream promoter sequences), and because the coding sequence remains unchanged. Alterations in 5′ structure between these different constructs are still possible, but the effect is probably diminished compared with the other studies and difficult to reliably assess computationally. We nevertheless observed that a third-order polynomial model again provided a better fit to the data than a first-order linear model (*R*^2^_adj_ = 0.37 and 0.316, respectively, *p* < 10^−10^ in both cases; figure 6*b*; electronic supplementary material, figure S10). We also observed that the intermediate binding quintile produced RTE values 85% higher than the weakest binding quintile, and observed a plateau or slight decrease in RTE for the strongest binding quintile of RBS sequences. This provides further support for our conclusion that translation efficiency is maximized at intermediate levels of aSD sequence complementarity and serves as an independent validation of our genome-scale findings. The large *R*^2^_adj_ values that we observed also provide strong empirical support for the hypothesis that some combination of error-prone mRNA structure prediction and error in the calculated RTE values strongly limit the observed *R*^2^_adj_ values in the genome-wide analyses, while the general trends and conclusions remain robust and are supported by this experimental dataset.

## 3. Discussion

Our work illustrates that there is a strong relationship between aSD sequence complementarity to the 5′ UTR of mRNAs and the translation of downstream endogenous genes. Specifically, we demonstrate that after accounting for the effects of mRNA structure: (i) aSD sequence complementarity to mRNA is predictive of translation efficiencies for endogenous genes within a relatively narrow window relative to the start codon, which can be empirically determined on a per-organism basis; (ii) slight changes in the putative aSD sequence significantly alter the statistical conclusions, allowing us to determine a data-driven definition of the optimal aSD sequence for each species; and (iii) intermediate aSD sequence complementarity maximizes the translation efficiency of downstream genes in all datasets that we encountered including well-controlled experimental data.

**Figure 7.**
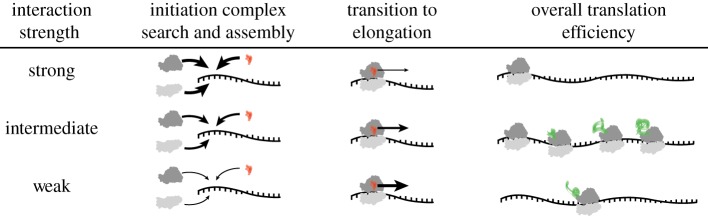
Model explaining why translation efficiency may be maximized for mRNAs with intermediate aSD sequence complementarity. The competing processes of initiation complex assembly and transition into elongation select for and against, respectively, strong aSD binding to mRNAs resulting in maximal translation efficiency for sequence with intermediate binding strength.

Our study complements and extends the experimental study of Vimberg *et al.* [44], who showed similar patterns of decreasing translation efficiency for experimentally manipulated genes with extended aSD sequence complementarity [44]. While it is possible that native sequences do not typically have strong sequence complementarity and that this effect would thus only apply to a small range of artificial gene constructs, we show here that a substantial number of genes from each genome actually fall within the regime decreased translation efficiency owing to the strength of their aSD sequence complementarity. Overall translation efficiency appears to be maximized at intermediate levels of complementarity between the aSD sequence and mRNA, possibly as a result of competing processes governing the efficiency of initiation complex assembly and the transition to translation elongation (figure 7)—as originally articulated by Komarova *et al.* [5,43–45]. Alternatively, rapid loading of ribosomes on a single mRNA may cause ribosomal queuing, and potentially result in premature termination or frame-shifting as ribosomes unproductively stall—thus decreasing overall ribosomal throughput on a given message [46]. More accurate experimental and computational protocols that limit sources of error and allow for more precise mapping of ribosome locations may fully resolve these and other issues.

Many previous bioinformatic and experimental studies either implicitly or explicitly assume a continual increase in translation efficiency with increasing aSD sequence complementarity [3,11,26]. One possible reason for this discrepancy is that many experiments may not observe a drop-off in efficiency at high levels of aSD sequence complementarity because they fail to access the full range of sequence diversity capable of binding to the 16S tail. We show here that mRNAs with perfect sequence complementarity to the core aSD sequence appear to translate just fine (figure 2*b*, linear fit). However, when considering the fact that sequence binding beyond the core aSD sequence appears to occur in all of these species, perfect complementary becomes detrimental as it begins to include base pairing to these flanking sequences.

Our goal here has not been to develop a comprehensive model to predict translation efficiencies measured by ribosome profiling, but rather to ask whether the sequence diversity and translation efficiency measurements for thousands of native genes can provide insights into the basic mechanisms of initiation. It is nevertheless surprising that the predictive power of the aSD::SD relationship is so low given that the aSD sequence is so highly conserved across nearly all bacterial species, and experimental investigations have seen large changes in protein output when modulating 5′ UTR sequence binding to the aSD sequence [3]. However, as we have stressed throughout, we note here again that our findings probably represent a lower bound on the predictive power of this interaction for several reasons. Genome-scale metrics are subject to both technical and biological noise, and translation efficiency as a metric will particularly suffer from this noise due to error-propagation. Further, mRNA folding around the start codon is known to exert a large effect on translation efficiencies and computationally predicted structures are rough approximations of the true mRNA structure [42]. It is thus reasonable to assume that these sources of noise contribute to lowering the expected ‘perfect’ correlations far below 1.0 as has been observed for other systems [10]. Despite these concerns, the underlying relationship that we observe is strong enough to show robust, statistically significant correlations in all datasets that we investigated. In the most controlled dataset that we analysed, a third-order model of aSD sequence complementarity explained roughly 40% of the observed variance in translation efficiency within an experimental system where the structure surrounding the start codon should be *relatively* similar across different constructs on account of the same coding sequence being expressed.

In addition to measurement noise and other caveats listed above, predicting the translation efficiency of endogenous genes poses a number of other unique challenges that contribute to low correlations. The location of transcription start sites relative to the start codon is variable, and experimentally measured 5′ UTRs are often shorter than 30 bases (and sometimes far longer). Further, a number of important genes such as ribosomal proteins are known to be regulated at the level of translation by various mechanisms that obscure statistical signal and which act in addition to the general patterns that we are trying to study. On top of all these limitations, we are also aware that translation efficiency may be modulated by differential elongation and termination in a non-trivial manner and that even within the realm of translation initiation other mechanisms such as the binding of ribosomal protein S1 may further modulate initiation efficiencies. Investigating the full range of possible contributions from each of these effects is far beyond the scope of our study, but doing so in the future will be valuable for better understanding translational regulation.

A better understanding of the rules governing translation initiation and translation efficiency stemming from this systems-biology approach has the practical potential to enhance our ability to design and engineer optimal protein expression systems for a host of biotechnological purposes. Particularly, orthogonal ribosome systems consisting of 30S subunits with altered aSD sequences (and corresponding mRNA sequence preferences) are an increasingly used tool in the synthetic biology community [47,48]. The effect that expression of these ribosomes has on endogenous genes governs their orthogonality, and predicting these effects based on the results that we show here may form an important part of rationally designing optimal systems that balance orthogonality against native genes and high expression of target genes [32].

Continued development and application of the ribosome profiling technique and associated technologies to diverse organisms will be critical for clarifying a number of outstanding questions in the field of translation and advancing our understanding of less well-understood species. While detailed experimental studies that systematically express and measure heterologous constructs remain the gold standard for studying sequence-based control of gene expression, we show here that genome-scale approaches combining RNA sequencing and ribosome profiling of native genes can provide valuable insights into these same mechanisms—making this approach particularly attractive for species with less established experimental protocols. Studying the sequence effects on translation in endogenous genes thus provides a valuable and complementary approach to long-standing experimental and bioinformatic investigations.

## 4. Material and methods

### 4.1. The data and relative translation efficiency

We downloaded ribosome profiling reads and corresponding RNA-sequencing reads for*E. coli*, *Caulobacter crescentus* and *Bacillus subtilis* [11,33,40]. We used the original researchers mapping of sequence reads to the respective genomes (.wig files) and removed genes with coverage below 25% in either the RNA-seq or ribosome profiling datasets in order to enrich for high-confidence measurements. We also removed any gene shorter than 30 codons as well as potentially misannotated genes with zero ribosome profiling reads to the first 10 nucleotides. For all remaining genes, we calculated translation efficiency for each gene as the RPKM in the Ribo-seq dataset divided by the RPKM in the RNA-sequencing dataset. We separately compiled two further datasets for *E. coli*, subjecting them to the same pipeline as above [37]. We settled on this approach as it is far more strict in data inclusion criteria than previous studies (which should partially limit noise in RTE measurements) while still providing reasonably large numbers of genes for analysis.

We further use two experimental datasets to independently validate our conclusions. The first from Taniguchi *et al.* [4] used single-cell distributions of protein counts to estimate the proteins produced per mRNA from fitted gamma-distributions of single-cell expression [4]. From the original dataset of 1018 genes we remove four from our analysis for quality control (i.e. coding sequences which are not a multiple of 3, do not have a ‘product’ annotation, contain internal stop codons, etc.). Because estimates for translation efficiency in this dataset were based on model fitting under the assumption of gamma-distributed protein concentrations, we analysed the subset of proteins (*n* = 717) for whom the probability of gamma fit was greater than 95%. For clarity, we maintain the label of RTE to describe these data but stress that their derivation is unrelated to ribosome profiling-based estimates of translation efficiency and that RTE in this context has a slightly different interpretation [4].

We also downloaded experimental data from recombinant gene expression in *E. coli* [22]. Each of 110 different ribosomal binding sites (RBSs) were characterized using FLOW-Seq (a method that combines fluorescence-activated cell sorting and high-throughput DNA sequencing) and can be described by their average protein levels across different promoters divided by the average mRNA levels (roughly equivalent to RTE when calculated for the same protein; from their initial data we exclude the ‘Dead-RBS’ construct because its short length is prohibitive to our analysis). Here we analyse this ‘mean.xlat’ data (as described in their supporting tables of [22]) as a measure of relative translation efficiency. As before, for ease of language, we continue to refer to this as RTE but note the slight differences in interpretation. Rather than subtracting out the effect of mRNA structure as in the previous datasets, we simply provide regressions on this raw data here because (i) the downstream gene is the same (and thus structure is mostly preserved between constructs), and (ii) each promoter will introduce slightly different sequences upstream of the RBS but their structural effects of this introduction should be accounted for in the averaging process.

### 4.2. Gene classification and quantification of aSD binding strength

All calculations of RNA folding were performed using the RNAfold method from ViennaRNA with default parameters [49]. Estimations of *cis*-structure were based on calculated folding energies for the −30 to +30 nt region relative to the start codon (‘A’, ‘T’, ‘G’ are bases +1, +2 and +3, respectively). RNA::RNA hybridizations were performed using the RNAcofold method with default parameters. For each gene, we iterated through all *x*-mers (where *x* is the length of the putative aSD sequence) upstream of the start codon in order to capture 14 hybridization events.

### 4.3. Operon predictions

We used predicted operons from the Database of Prokaryotic Operons [50]. From these data tables, we classified each gene according to whether it is predicted to occur first within a transcription unit or whether another gene precedes it within a transcription unit, regardless of the distance.

### 4.4. Statistics and code sharing

All code used to perform translation efficiency measurements, as well as all statistics were written using custom scripts in Python that are included in the electronic supplementary material. All regression models and statistics (including *R*^2^, *R*^2^_adj_ and AIC) were performed using the statsmodels package from Python; reported *p*-values in all regressions are based on the *F*-test. Code and necessary data to recreate figures are available at https://github.com/adamhockenberry/OpenBiology_2016.

## Supplementary Material

Supporting Information
