# Supplementary material for: Leveraging genome-wide datasets to quantify the functional role of the anti-Shine–Dalgarno sequence in regulating translation efficiency
Source: Open Biol. 2017 Jan 18;7(1):160239. doi: 10.1098/rsob.160239 (PMC5303271; doi:10.1098/rsob.160239)
Supplement: Supporting Information [file rsob160239supp1.pdf]

Supporting Information

Leveraging genome-wide datasets to quantify the functional role of the anti-Shine-Dalgarno sequence in regulating translation efficiency

Adam J. Hockenberry[1,2], Adam R. Pah[3,4], Michael C. Jewett[1,2,5,*], and Luís A. Nunes Amaral[2,3,6,*]

[1]Interdisciplinary Program in Biological Sciences, Northwestern University, Evanston, IL, 60208, USA
[2]Department of Chemical and Biological Engineering, Northwestern University, Evanston, IL, 60208, USA
[3]Northwestern Institute on Complex Systems, Northwestern University, Evanston, IL, 60208, USA
[4]Kellogg School of Management, Northwestern University, Evanston, IL, 60208, USA
[5]Chemistry of Life Processes Institute, Northwestern University, Evanston, IL, 60208, USA
[6]Department of Physics and Astronomy, Northwestern University, Evanston, IL, 60208, USA

* To whom correspondence should be addressed. Tel: 847-491-7850; Email: amaral@northwestern.edu; Correspondence may also be addressed to m-jewett@northwestern.edu

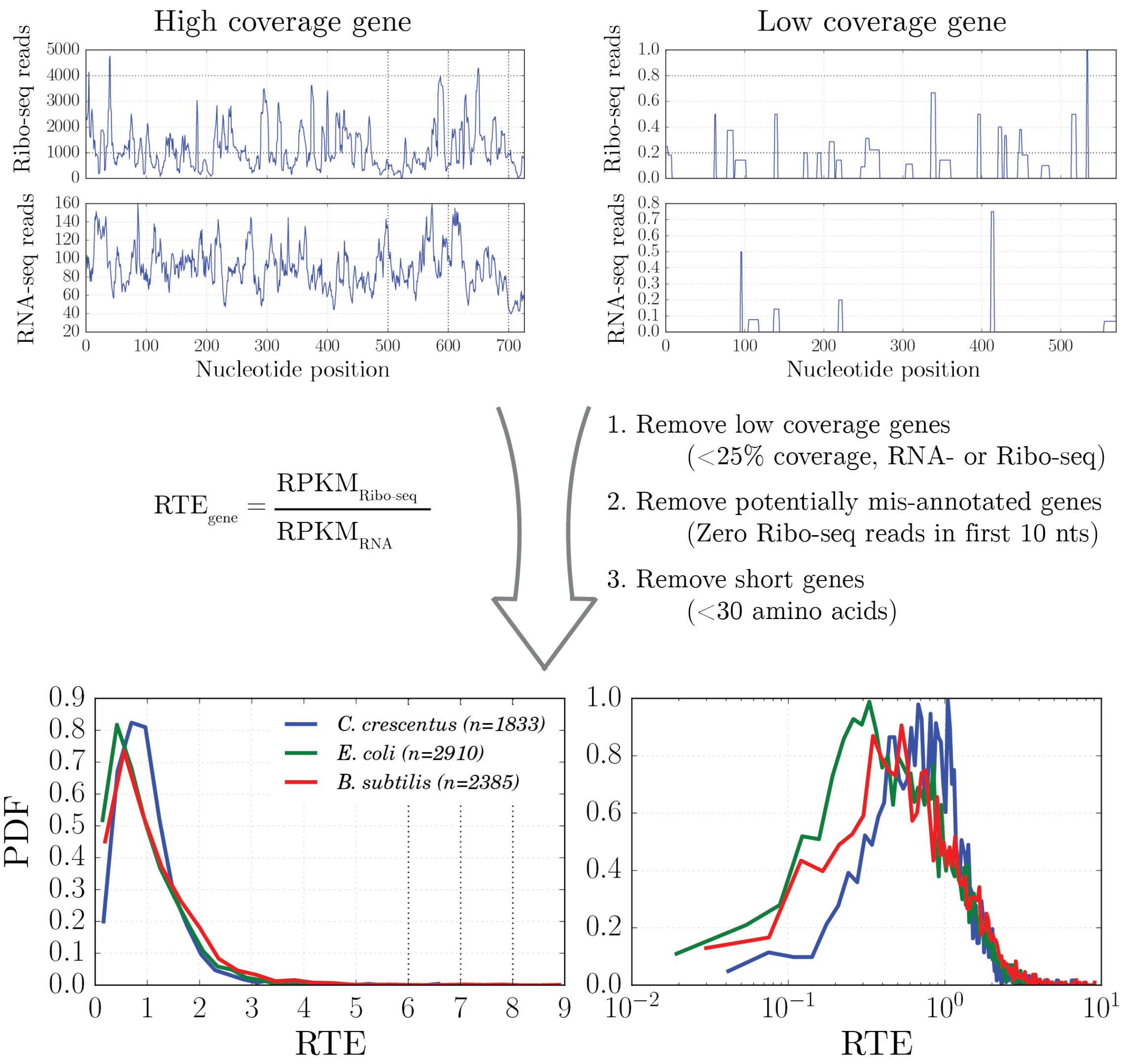

Figure S1: Example gene profiles showing mapped RNA- and Ribo-seq reads that are used as input to calculate RTE. Our pipeline first removes a subset of the total genes based off of coverage, annotation, and length requirements resulting in RTE measurements for 2910, 1833, and 2385 genes in *E. coli, C. crescentus* and *B. subtilis*. Distributions of the RTE values on normal and log-scale show that RTE is approximately log-normally distributed and comparable between the three datasets studied.

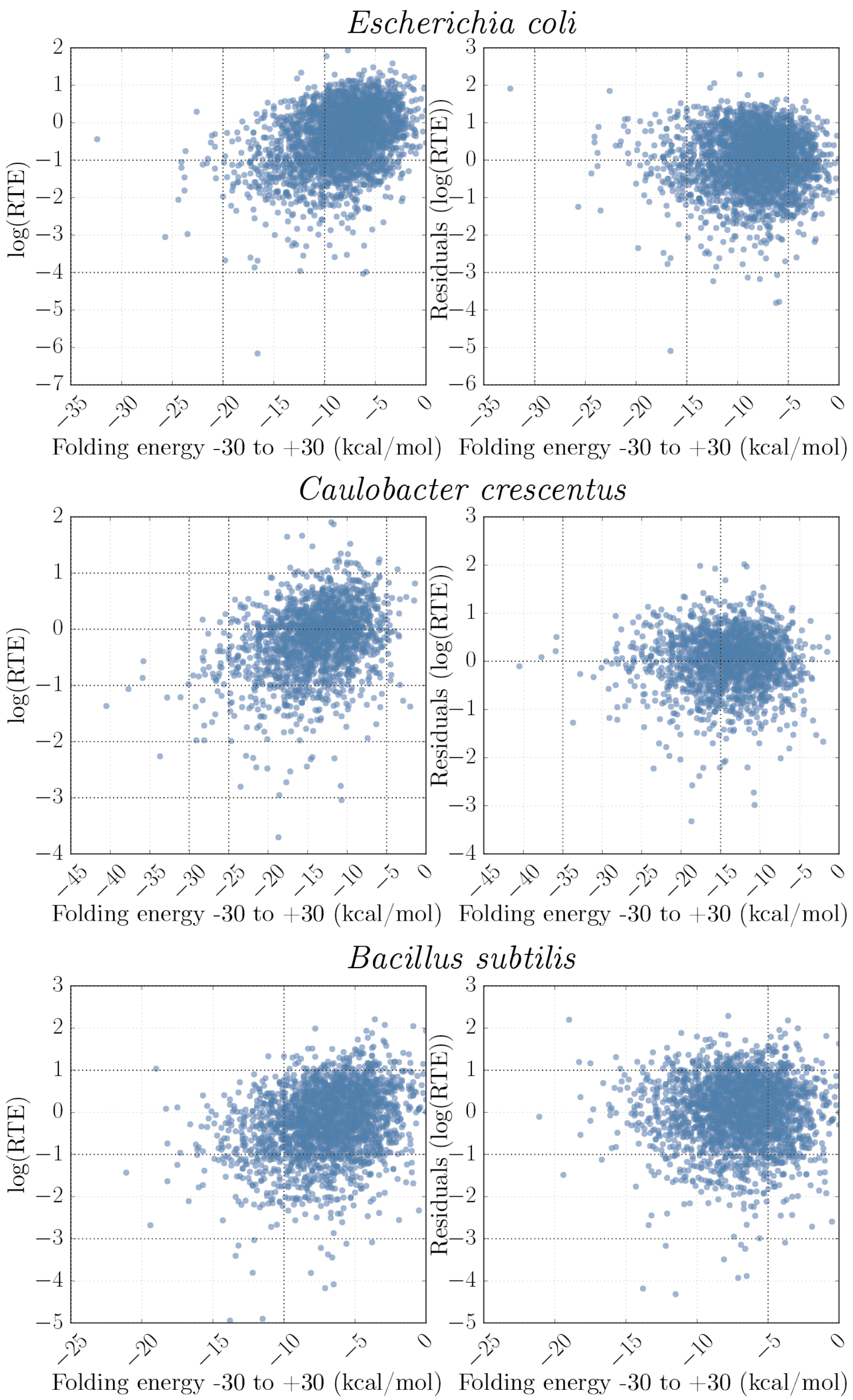

Figure S2: Correlation between the free energy of RNA folding around the start codon (-30 to +30) and log(RTE) for three different organisms studied (left, R^2 =0.13, 0.10, 0.08 for *E. coli, C. crescentus,* and *B. subtilis* respectively; for all cases $p<10^{-43}$). For RTE in the main text we utilize the residuals from the best fitting linear model based off this regression for each organism, effectively removing the influence of mRNA structure on RTE (right).

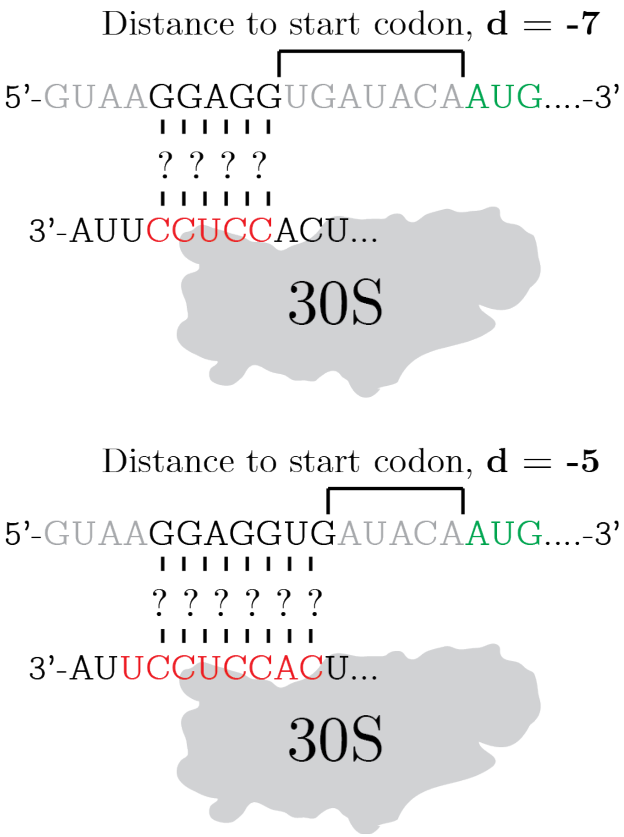

Figure S3: Extended illustration of our numbering scheme for distance. This example highlights that distance is not absolute and, rather, is calculated relative to the aSD sequence being considered. For the same mRNA sequence (top and bottom), the distance decreases by two due to the fact that the example in the bottom extends the hypothetical aSD sequence 2 bases in the 5' direction. Numbering is always relative to the 5' end of the aSD sequence so varying this sequence. Other numbering systems to compare between different putative aSDs will all suffer from this problem unless an absolute point is used as an anchor (such as the middle U of 5'-CCUCC-3'). We opted for our scheme because we feel that, at the conclusion of the process, our scheme is simpler to interpret for a given aSD sequence.

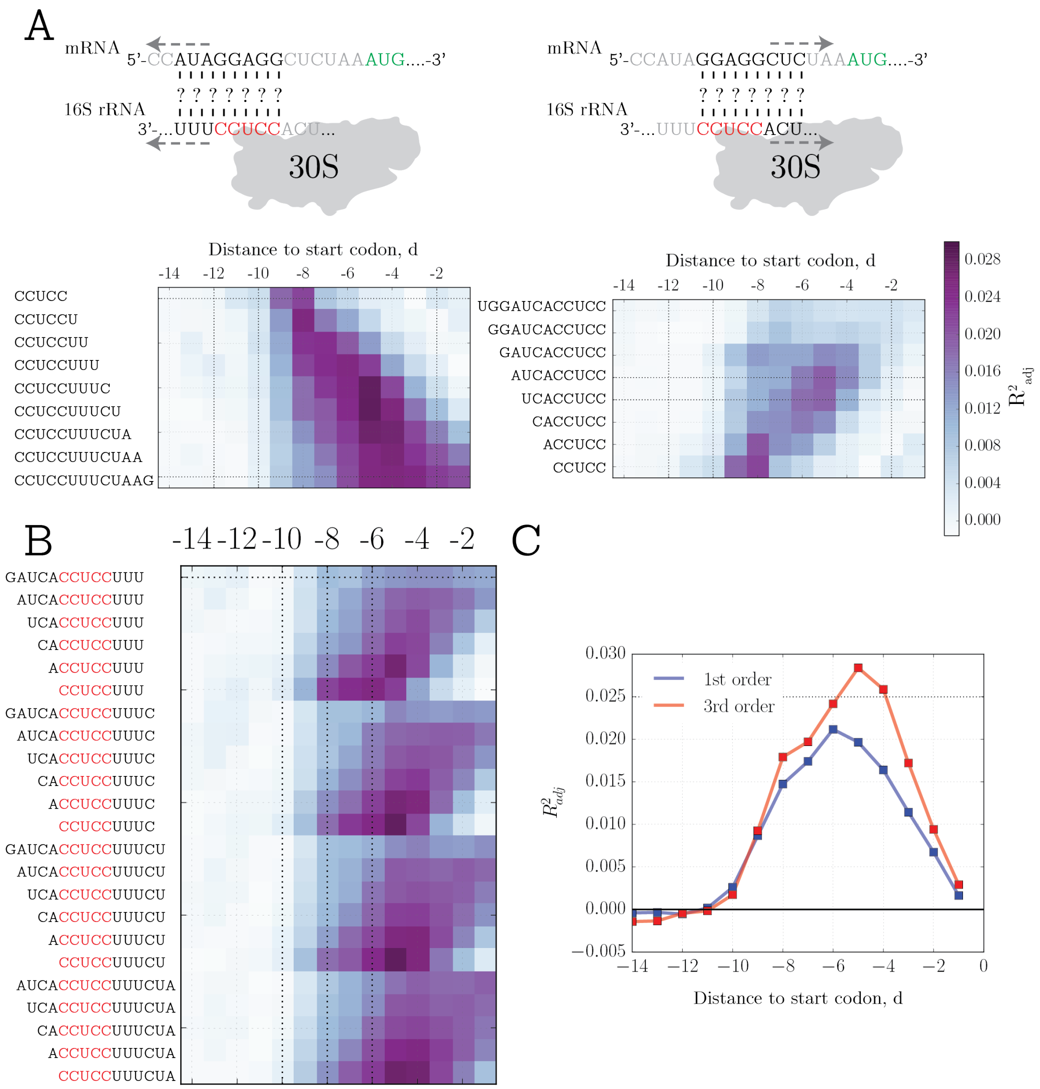

Figure S4 (related to main text Fig. 3): A) R^2_{adj} from the 3rd order model at different distances to the start codon and various 3' and 5' extensions to the core aSD for *C. crescentus*. B) Combination of best fitting putative aSDs from (A) to determine the optimal aSD sequence. C) Comparison of R^2_{adj} between the 1st and 3rd order polynomial models from best performing aSD sequence.

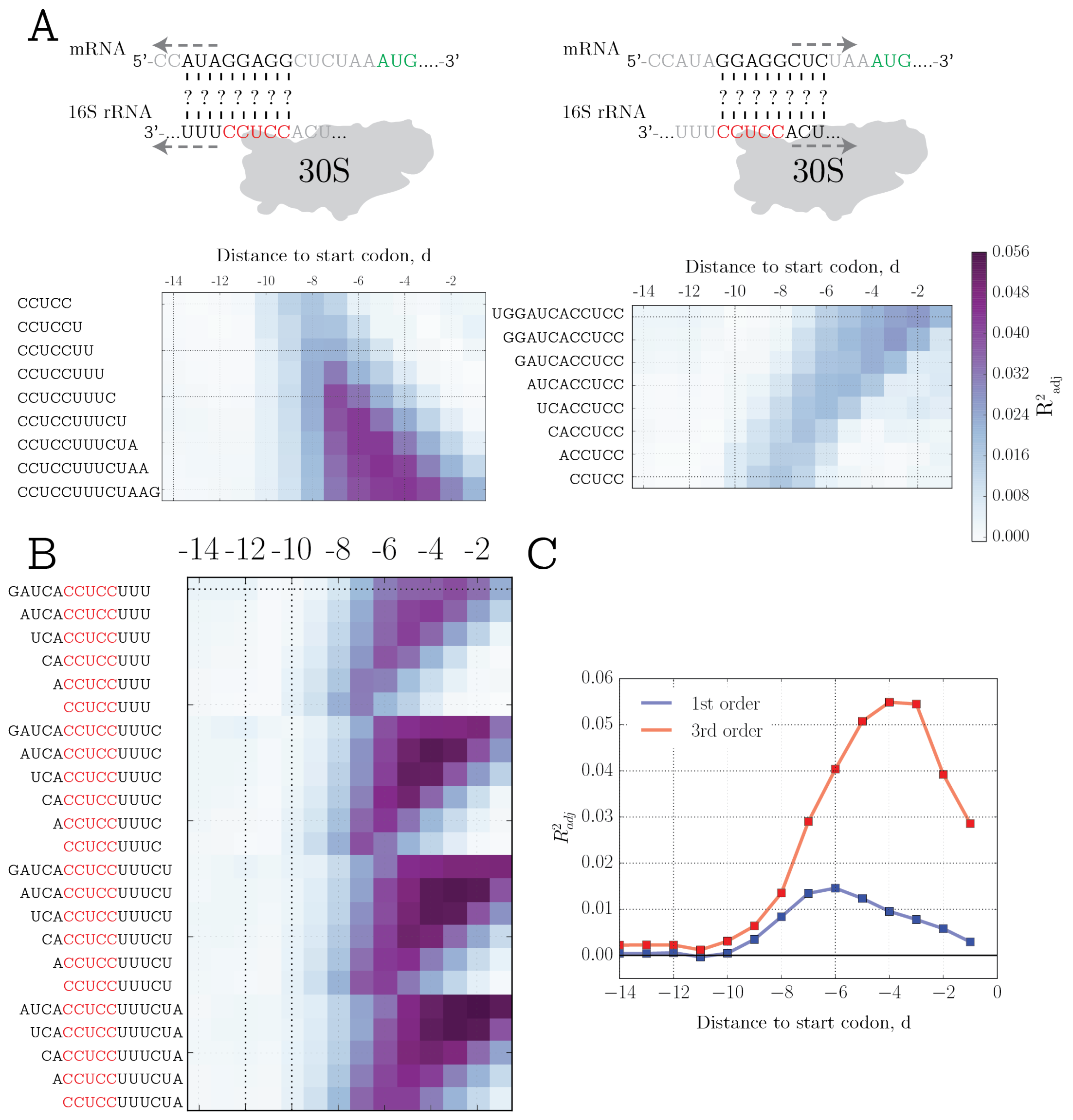

Figure S5 (related to main text Figure 3): A) R^2_{adj} from the 3rd order model at different distances to the start codon and various 3' and 5' extensions to the core aSD for *B. subtilis.* B) Combination of best fitting putative aSDs from (A) to determine the optimal aSD sequence. C) Comparison of R^2_{adj} between the 1st and 3rd order polynomial models from best performing aSD sequence.

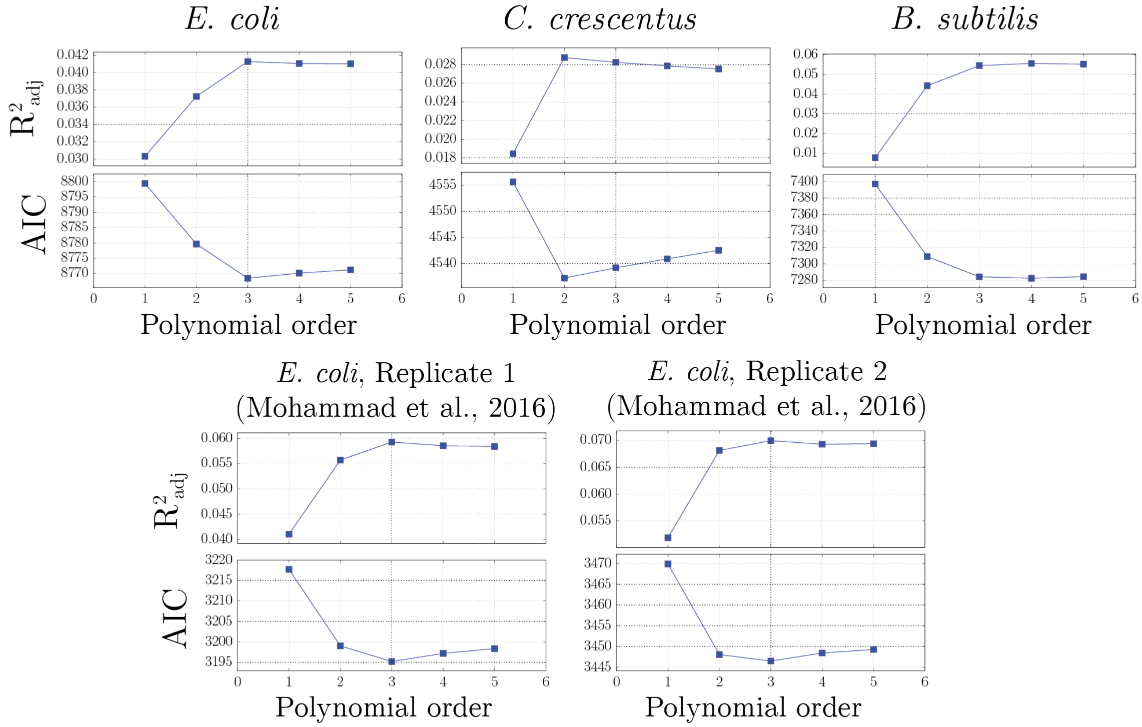

Figure S6 (related to main text Figs. 4 & 5): For *E. coli* data (left), given the optimal distance and aSD parameters, we show the effect of increasingly complex polynomial fits on the R^2_{adj} (top) and Akaike Information Criterion (AIC)(bottom), two statistical methods commonly used for model selection. Both metrics penalize models with increasing parameter number through different statistical means in order to prevent over-fitting; the best model, according to the R^2_{adj}, should be the one that maximizes this metric while for the AIC the best model should minimize this value. The data are also shown for *C. crescentus* (center) and *B. subtilis* (right) data, in all cases this data was calculated using the optimal aSD and spacing values indicated in Fig. 4 of the main text for each organism. Bottom row shows this same data for *E. coli* based on the data used in Fig. 5 of the main text, from Mohammad *et al*. (2016).

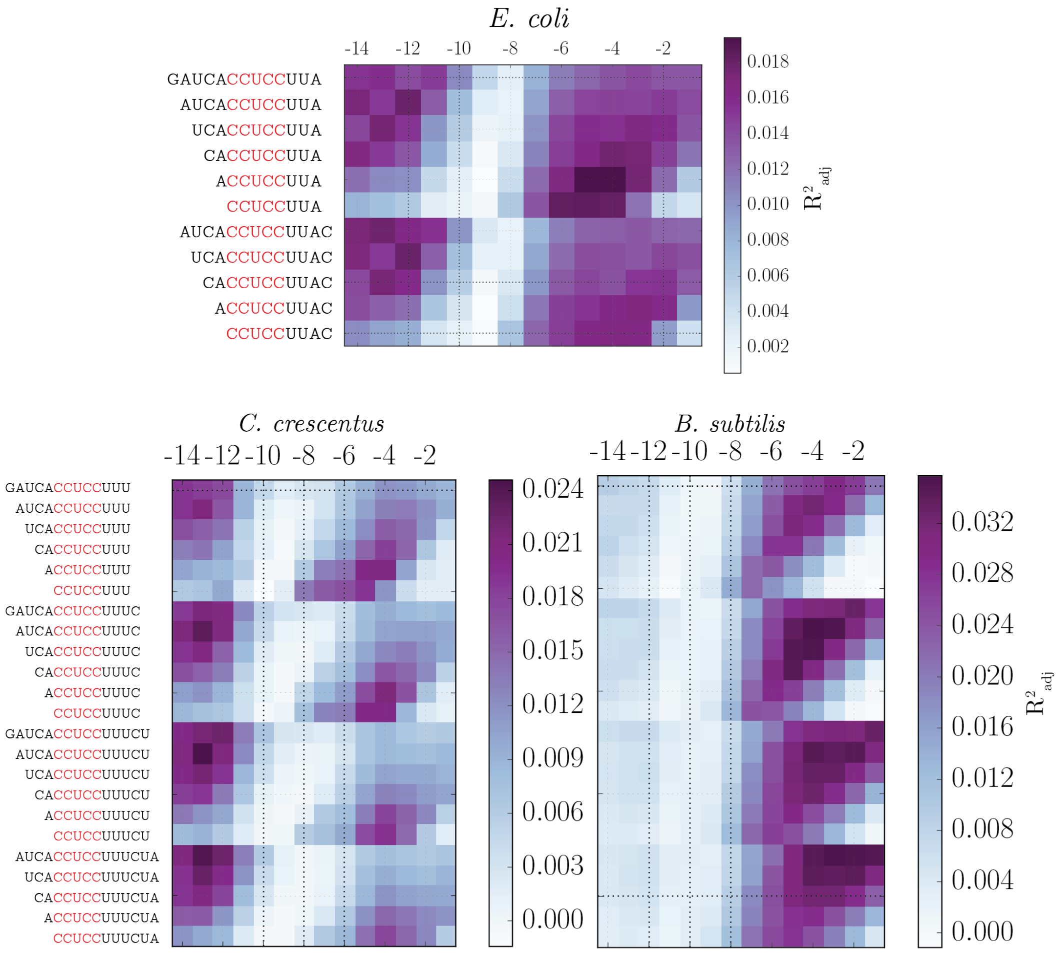

Figure S7 (as in main text Fig. 3 and Supporting Figures S4 & S5). Whereas the previous results display correlations between aSD sequence complementarity and residual *RTE* values (calculated by removing the effect of predicted mRNA structure), here we repeated our algorithm to choose the best fitting aSD sequence and distance parameters given the raw log-transformed *RTE* values. For each of the three different organism datasets displayed, the qualitative conclusions are similar with decreases in the overall magnitude and significance of the observed effect but clear peaks for particular aSD and distance combinations which closely align with the conclusions in the main text. We attribute the increasing significance on the left side of the *E. coli* and *C. crescentus* data to the fact that the aSD sequence complementarity is likely measuring GC content in this region and thus mRNA structure by proxy.

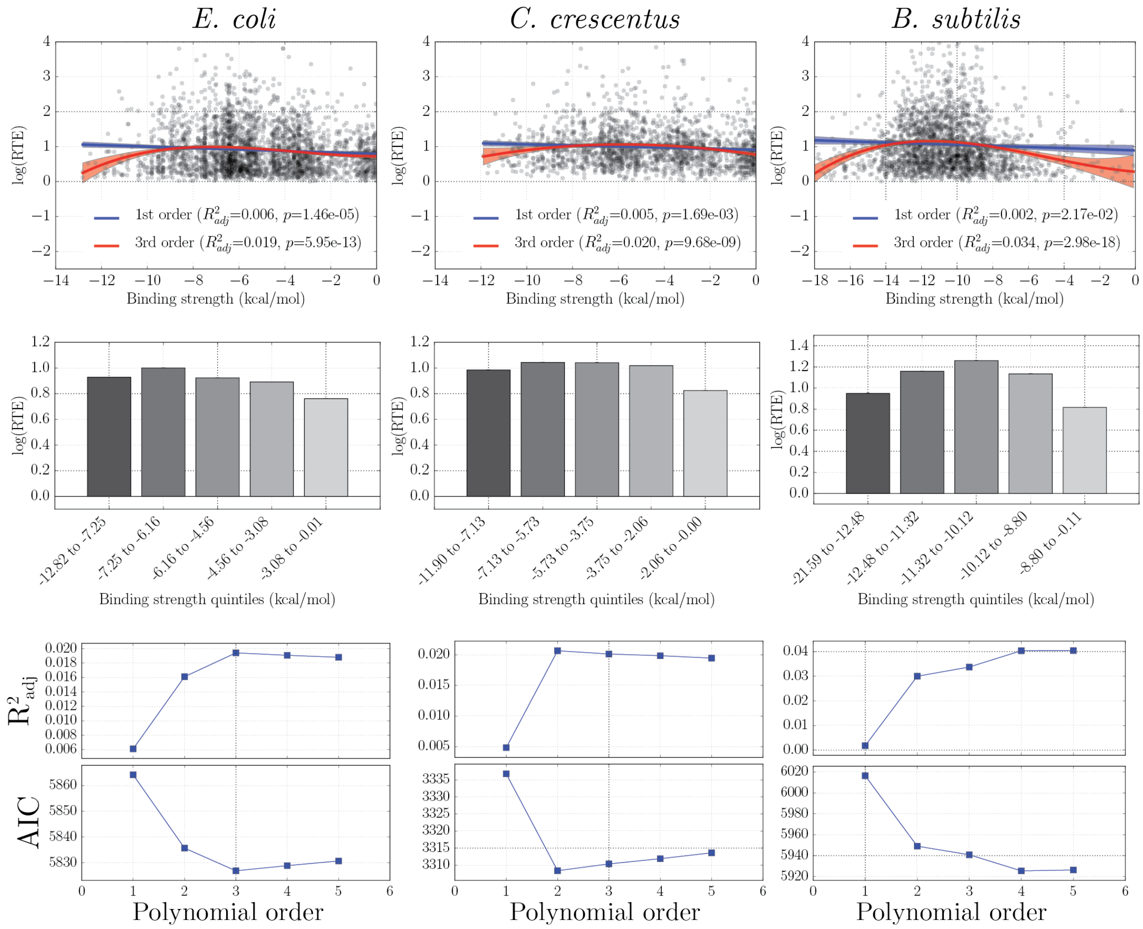

Figure S8 (as in main text Fig. 4): We repeated our analysis of the relationship between aSD sequence complementarity and translation efficiency by looking at the log-transformed *RTE* values (rather than the residual *RTE* values calculated by removing the effect of predicted mRNA structure) for the best fitting aSD sequence and distance parameters discovered in the main text. In all cases, a 3rd order model is highly statistically significant, and fits the data better than a 1st order model. Further, quintile bins again show that the strongest binding quintile of genes for all datasets exhibits reduced *RTE* values.

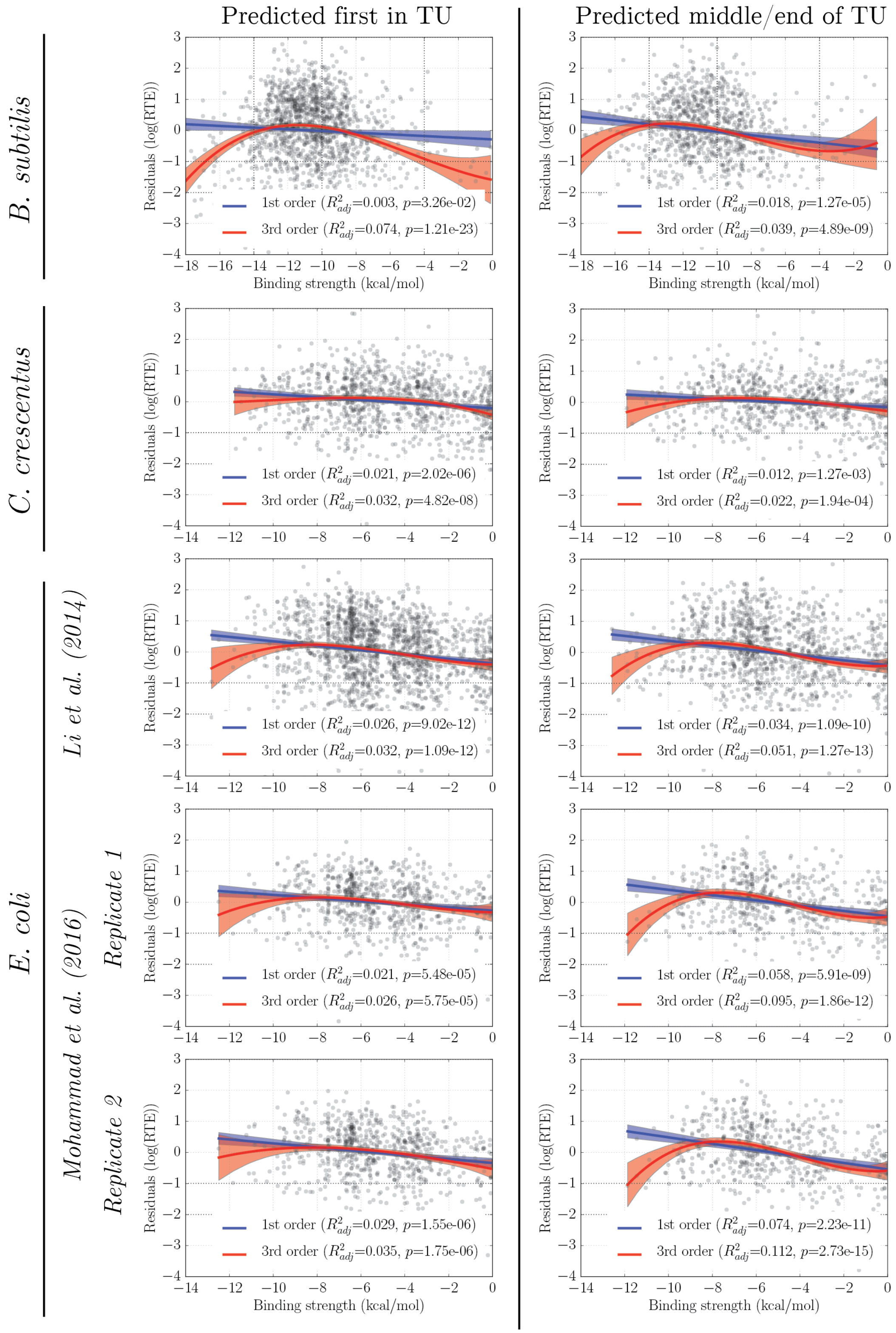

Figure S9: Robustness of the results with respect to gene position within operons for the indicated datasets. aSD sequences and distances for each dataset are as in Figs. 4 and 5 of the main text.

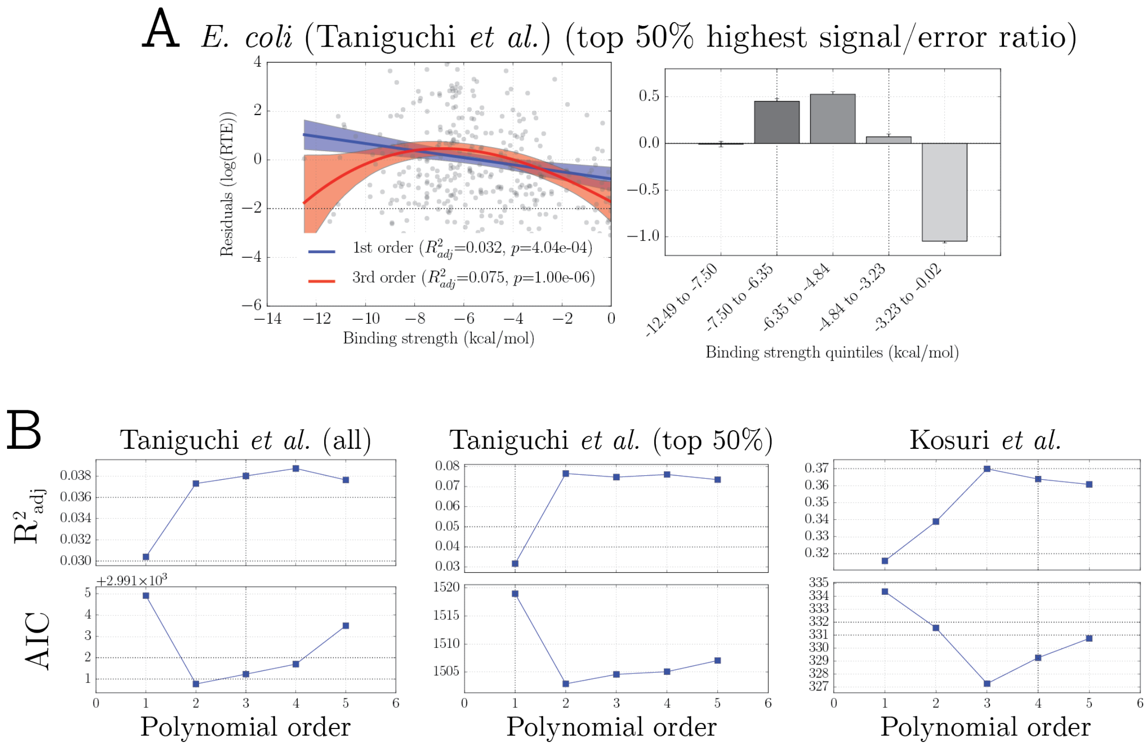

Figure S10: A) As in main text Figure 6A, we analyzed the Taniguchi *et al.* dataset but here restrict the analysis to the top 50% genes in this dataset with the highest signal/error ratio and observe similar trends to Figure 6A but with stronger predictive power of the underlying polynomial model as assessed by the R^2_{adj} value. B) The relevant AIC and and R^2_{adj} values for the indicated datasets from Figure 6.
